# Comparison of Single Axillary vs. Dual Arterial Cannulation for Acute Type a Aortic Dissection: A Propensity Score Matching Analysis

**DOI:** 10.3389/fcvm.2022.809493

**Published:** 2022-02-23

**Authors:** Yi Chang, Hongyuan Lin, Xiangyang Qian, Hongwei Guo, Cuntao Yu, Xiaogang Sun, Bo Wei, Qiong Ma, Yizhen Wei, Yi Shi

**Affiliations:** Department of Vascular Surgery, National Center for Cardiovascular Diseases, Fuwai Hospital, Cardiovascular Institute, Chinese Academy of Medical Science & Peking Union Medical College, Beijing, China

**Keywords:** aortic dissection, total arch replacement, frozen elephant trunk, cannulation, propensity adjustment

## Abstract

**Background:**

The optimal arterial cannulation site for acute aortic dissection repair is unclear, especially for complex arch surgery. Axillary artery cannulation is widely accepted but adding femoral artery cannulation to it was considered to potentially improve perfusion and early outcomes. To clarify this point, a comparison of perioperative outcomes for these two different cannulation strategies was conducted regarding the pathological features of dissection.

**Methods:**

From January 2010 to December 2019, 927 consecutive patients underwent a total arch replacement combined with frozen elephant trunk for acute type A aortic dissection. The data, including detailed pathological features, were retrospectively collected and analyzed. Propensity score matching and multivariate logistic regression analysis were used for adjusting confounders that are potentially related to the outcome.

**Results:**

A total of 523 patients (56.3%) accepted a dual arterial cannulation (DAC group), and 406 patients (43.7%) received a single axillary artery cannulation (SAC group). In total, 388 pairs of patients were well-matched. Whether before or after adjusting the preoperative characteristics by matching, there were no significant differences in operative mortality (6.7 vs. 5.4%, *P* = 0.420 before matching; 5.4 vs. 5.4%, *P* = 1 after matching), stroke (6.7 vs. 5.4%, *P* = 0.420 before matching; 6.4 vs. 5.2%, *P* = 0.435 after matching), spinal cord injury (5 vs. 5.7%, *P* = 0.640 before matching; 5.4 vs. 5.7%, *P* = 1. After matching), and acute renal failure requiring dialysis (13.8 vs. 9.6%, *P* =0.050 before matching; 12.6 vs. 9.5%, *P* = 0.174) between the two groups. Dual arterial cannulation was not an independent protective factor of operative mortality (odds ratio [OR] 1.01, 95% confidence interval [CI] 0.55–1.86), stroke (OR 1.17, 95% CI 0.65–2.11), spinal cord injury (OR 1.17, 95% CI 0.65–2.11), and acute renal failure requiring continuous renal replacement therapy (CRRT) (OR 1.24, 95% CI 0.78–1.97) after adjusting for confounding factors by multivariable logistic regression analysis. In the subgroup analysis, no advantage of dual arterial cannulation was found for a particular population.

**Conclusions:**

Single axillary artery cannulation was competent in the complex arch repair for acute aortic dissection, presenting with a satisfactory result as dual arterial cannulation. Adding femoral artery cannulation was necessary when a sufficient flow volume could not be achieved by axillary artery cannulation or when a lower limb malperfusion existed.

## Introduction

Nowadays on China, there is a tendency to perform a total arch replacement (TAR) with frozen elephant trunk (FET) as a standard approach for acute type A aortic dissection (ATAAD), involving arch or more distally. The TAR combined with FET is more invasive and complex so that more evidence is needed to prove the optimal management for each step of this procedure. Regarding the arterial cannulation site, although the latest expert consensus of AATS (1) recommended kinds of options, controversy always exists and high-level evidence is absent. In the past years, we had attempted to apply a dual arterial cannulation to reduce the malperfusion-related mortality and morbidities by extrapolation based on clinical experience. We sought to compare dual arterial cannulation and single axillary artery cannulation on early outcomes by rigorous statistical analysis.

## Materials and Methods

### Study Cohort

The institutional database of aortic dissection was retrospectively reviewed. All consecutive patients who were surgically treated for ATAAD using TAR and FET from January 2010 to December 2019 were included. The patients, who were not appropriate for axillary cannulation (for example, the vessel was too thin or was dissected), accepted a single femoral cannulation and they were excluded from this study (flowchart of enrollment can be seen in Supplementary Figure I). The Ethics Committee of Fuwai Hospital (Beijing, China) approved this retrospective study, and the need for informed consent was waived. The data of demographics, clinical features, imaging materials, surgical characteristics, and postoperative outcomes were collected.

### Institution-Specific Definition

To identify the special conditions and scenarios that would influence the progress and outcome of ATAAD, we defined some institution-specific terms to describe them. Hemodynamic instability was defined as persistent pre-anesthesia hypotension (systolic blood pressure <90 mmHg) that is preoperative for any reason. Innominate artery stenosis (IAS) was defined as severe stenosis of the true lumen caused by thrombus compression. Innominate artery, originating from the false lumen (IA-FL), as the name suggests, referred to the detachment or complete avulsion of its orifice. All the malperfusion mentioned in this study referred to the obviously disrupted blood flow of main branches of the aorta with radiographical evidence. Because superior mesenteric artery malperfusion had particular importance in ATAAD, it was identified and categorized into three types according to prior studies (2–4). Similarly, lower limb malperfusion was determined as mentioned above. Selective antegrade cerebral perfusion duration usually started from distal circulatory arrest initiation (at this moment three brachiocephalic arteries were clamped) until the anastomosis of the left common carotid artery was completed.

### Clinical Endpoints

Operative mortality, stroke, spinal cord injury, and acute renal failure requiring continuous renal replacement therapy (CRRT) were primary endpoints of interest. Operative mortality was defined as any death, regardless of cause, occurring within 30 days after surgery in or out of the hospital, and after 30 days during the same hospitalization subsequent to the operation. For stroke, spinal cord injury, and renal failure, only the new onsets were considered as complications. Spinal cord injury included permanent or transient paresis and paraplegia. All the outcomes were defined using the Society of Thoracic Surgeons definitions (see http://www.sts.org/sts-national-database/database-managers/adultcardiac-surgery-database/data-collection).

### Operative Methods

All procedures were implemented by a median sternotomy and cardiopulmonary bypass (CPB). Eight surgeons finished these operations (QXY, GHW, YCT, SXG, WB, MQ, WYZ, and SY). Arterial cannulation was manipulated as follows: (1) right axillary artery: it was exposed by subclavian incision and a purse-string suture was made on it, then a cannula (Bio-Medicus, Medtronic, Minneapolis, MN, USA) with a tapered core was directly inserted through a small incision inside the purse-string; and (2) femoral artery: it was exposed by an incision parallel to the inguinal ligament and cannulated in the same manner as described above. We used axillary artery cannula according to body weight and axillary artery size, cannula size ranged from 17Fr- 21Fr, median size was 19 Fr. We usually selected the 19 Fr−21Fr cannula for the femoral artery.

After distal anastomosis of the arch was accomplished, the femoral artery cannula was removed and the perfusion conduit was shifted to 1 limb of the 4-branch prosthetic graft. In our institute, dual arterial cannulation (right axillary artery+ femoral artery) was individually selected, mainly according to the preference and judgment of the surgeon. In rare cases, we selected femoral artery cannulation as a supplement to axillary conduit owing to insufficient flow volume. Venous return was achieved *via* a two-staged cannula placed in the right atrium or cannulas in the vena cava as appropriate. Indication and technique of performing TAR combined with FET (CRONUS; MicroPort, Shanghai, China) in our institution were similar to those described by Sun et al. (5) (More detailed information about indication and technique was available in Supplementary Material Expanded Methods). Briefly, we used a 4-branch prosthetic graft to replace the arch and a stented graft that is deployed anterogradely into a descending aorta to isolate the false lumen. During FET deployment and distal arch anastomosis, hypothermia circulatory arrest (HCA) and unilateral selective cerebral perfusion were applied. The target nasopharyngeal temperature during HCA was 18–25°C in different periods.

### Statistical Analysis

Data were presented as mean and standard deviation for a continuous data conforming to the normal distribution and as number (%) for categorical data. The mean of two continuous normally distributed variables was compared by independent samples student *t*-test. Comparison of categoric variables between groups was analyzed by the likelihood ratio of Chi-square test or Fisher's exact test. The continuous variable that did not conform to a normal distribution was demonstrated as the median and interquartile range (IQR) and were compared by Wilcoxon signed-rank test. The missing quantitative data were filled with mean and the qualitative data were filled with maximum frequency.

Propensity score matching was applied to achieve a balanced exposure between groups at baseline (i.e., minimal confounding). The probability of each patient having a dual arterial cannulation (i.e., the propensity score) was calculated using a logistic regression model. Covariates included age, gender, preoperative comorbidities, and pathologic features of dissection were adjusted (see Supplementary Table I). Patients were then matched one-to-one using the Nearest-neighbor matching and caliper width of 0.1 of the standard deviation of the logit of the propensity score. After propensity score matching, a comparison of continuous data conforming to normal distribution between groups was analyzed by paired *t*-test. Paired Chi-square test was used to compare multiple categorical variables between the two groups, statistics and the *P* value of the symmetric test were adopted. The McNemar test was used to compare binary variables between the two groups. To investigate the influence of cannulation strategy on primary outcomes and to avoid a large deviation result, three multivariate logistic regression models adjusting to different confounders were constructed. Confounders were determined according to our clinical experience and previous studies (6, 7). Stratified analysis was conducted to identify whether a dual arterial cannulation had an advantage over a single axillary artery cannulation for a particular population. Statistical significance was denoted by *P* values < 0.05. The statistical analyses were conducted by SAS (version 9.4, SAS Institute Inc., Cary, North Carolina, America).

## Results

### Baseline Characteristics

Between January 2010 and December 2019, a total of 1,119 patients suffering from ATAAD were admitted emergently for undergoing TAR and FET, whereas 185 met the criteria for exclusion (Supplementary Figure I). Of this total, 929 patients comprised the interest cohort.

The mean age of the patient was 46.8 ± 10.1years, with a male preponderance (81.5%). A total of 523 patients (56.3%) accepted dual arterial cannulation (DAC group), and 406 patients (43.7%) received single axillary artery cannulation (SAC group). The preoperative serum creatinine level was significantly higher in the DAC group. Entry tear locating in ascending aorta was more common in the DAC group (69.4 vs. 60.3%, *P* = 0.009), on the other hand, the patients in the SAC group were more likely to have entry tear in the arch (24.1 vs. 33.0%, *P* = 0.009). The proportion of unilateral cerebral malperfusion was significantly higher in the DAC group (6.7 vs. 2.5%, *P* = 0.004). Other preoperative features were similarly distributed between the two groups. The baseline characteristics were shown in Table 1. For the whole cohort, 667 (71.8%) patients received a supracoronary aortic replacement. Bentall procedure, David procedure, and aortic valve replacement were carried out in 241 (25.9%), 9 (1.0%), and 12 (2.3%) patients, respectively. A total of 113 (12.2%) patients underwent a coronary artery bypass grafting for coronary artery disease or coronary artery involvement. Femoral and carotid artery bypass operations were implemented to rescue severely dissected vessels from occlusion caused by a thrombus compression. These bypass operations were performed in 32 (3.4%) and 15 (1.6%) patients. A similar proportion of concomitant procedures was found in the two groups. For patients undergoing dual arterial cannulation, CPB duration, X-clamp duration, and SCP, duration were all longer than those in the SAC group (204.8 ± 62.7 vs. 182.7 ± 72.6 min, 112.9 ± 31.9 vs. 102.4 ± 35.3 min, 29.5 ± 8.7 vs. 23.9 ± 7.0 min, *P* < 0.001). The nasopharyngeal temperature in the DAC group was significantly lower than in the SAC group (21.6 ± 3.8 vs. 22.4 ± 3.2°C, *P* = 0.004). Operative characteristics were demonstrated in Table 2. Altogether, 388 pairs of patients were matched using a propensity score matching. It was demonstrated that well-balanced absolute standardized differences between the two groups were achieved regarding the baseline characteristics (Supplementary Table II). After matching, there was no significant difference in terms of baseline characteristics between the 2 groups, although CPB duration, X-clamp duration, SCP duration, and nasopharyngeal temperature were still significantly different.

**Table 1:** Baseline characteristics.

	**Unmatched**			***P***	**Missing**	**Matched**			***P***
**Variable**	**Overall** ***n =* 929**	**DAC** ***n =* 523**	**SAC** ***n =* 406**			**Overall** ***n =* 776**	**DAC** ***n =* 388**	**SAC** ***n =* 388**	
Age, year ($\bar{X}$ ± SD)	46.8 ± 10.1	47.3 ± 10.2	46.2 ± 9.8	0.09	0 (0.0)	46.4 ± 10.0	46.4 ±10.1	46.3 ± 9.8	0.92
Male (*n*, %)	757 (81.5)	424 (81.1)	333 (82.0)	0.71	0 (0.0)	633 (81.6)	316 (81.4)	317 (81.7)	0.92
BMI, Kg/m^2^ ($\bar{X}$ ± SD)	26.4 ±4.1	26.6 ± 4.1	26.2 ± 4.0	0.17	2 (0.2)	26.2 ± 4.0	26.2 ± 3.9	26.2 ± 4.1	0.91
HT (*n*, %)	589 (63.4)	327 (62.5)	262 (64.5)	0.53	0 (0.0)	497 (64.0)	249 (64.2)	248 (63.9)	0.94
CAD (*n*, %)	58 (6.2)	37 (7.1)	21 (5.2)	0.23	0 (0.0)	43 (5.5)	22 (5.7)	21 (5.4)	0.88
AF (*n*, %)	8 (0.9)	3 (0.6)	5 (1.2)	0.31*	0 (0.0)	6 (0.8)	3 (0.8)	3 (0.8)	1.00
DM (*n*, %)	22 (2.4)	10 (1.9)	12 (3.0)	0.30	0 (0.0)	21 (2.7)	9 (2.3)	12 (3.1)	0.66
Marfan syndrome (*n*, %)	68 (7.3)	38 (7.3)	30 (7.4)	0.94	0 (0.0)	59 (7.6)	30 (7.7)	29 (7.5)	0.89
Previous stroke (*n*, %)	35 (3.8)	22 (4.2)	13 (3.2)	0.42	0 (0.0)	22 (2.8)	9 (2.3)	13 (3.4)	0.50
CRI (*n*, %)	7 (0.8)	3 (0.6)	4 (1.0)	0.71*	0 (0.0)	6 (0.8)	3 (0.8)	3 (0.8)	1.00
Scr, μmol/L ($\bar{X}$ ± SD)	99.09 ± 40.80	102.09 ± 46.14	95.24 ± 32.35	0.008	2 (0.2)	98.80 ± 41.84	102.55 ± 49.27	95.04 ± 32.40	0.012
Previous heart surgery (*n*, %)				0.20	0 (0.0)				1.00
No	895 (96.3)	502 (96.0)	393 (96.8)			757 (97.6)	380 (97.9)	377 (97.2)	
TEVAR	12 (1.3)	9 (1.7)	3 (0.7)			0 (0.0)	0 (0.0)	0 (0.0)	
AVR	9 (1.0)	3 (0.6)	6 (1.5)			7 (0.9)	3 (0.8)	4 (1.0)	
Others	13 (1.4)	9 (1.7)	4 (1.0)			12 (1.5)	5 (1.3)	7 (1.8)	
EF, % ($\bar{X}$ ± SD)	60.2 ± 4.3	60.1 ± 4.5	60.4 ± 4.0	0.18	4 (0.4)	60.4 ± 4.1	60.4 ± 4.2	60.4 ±4.0	0.90
AR>moderate (*n*, %)	110 (11.8)	70 (13.4)	40 (9.9)	0.09	1 (0.1)	74 (9.5)	34 (8.8)	40 (10.3)	0.46
Hemodynamic instability (*n*, %)	47 (5.1)	31 (6.0)	16 (4.1)	0.18	0 (0.0)	29 (3.7)	13 (3.4)	16 (4.2)	0.58
Tamponade	10 (1.1)	4 (0.8)	6 (1.5)	0.30		6 (0.8)	3 (0.7)	4 (1.0)	0.70
Entry tear (*n*, %)				0.009	0 (0.0)				0.68
aAO	608 (65.4)	363 (69.4)	245 (60.3)			483 (62.2)	243 (62.6)	240 (61.9)	
Arch	260 (28.0)	126 (24.1)	134 (33.0)			236 (30.4)	114 (29.4)	122 (31.4)	
DTA	61 (6.6)	34 (6.5)	27 (6.7)			57 (7.3)	31 (8.0)	26 (6.7)	
Extent (*n*, %)				0.14*	0 (0.0)				0.29
To arch	60 (6.5)	41 (7.8)	19 (4.7)			46 (5.9)	29 (7.5)	17 (4.4)	
To DTA	5 (0.5)	3 (0.6)	2 (0.5)			4 (0.5)	2 (0.5)	2 (0.5)	
To distal AA	864 (93.0)	479 (91.6)	385 (94.8)			726 (93.6)	357 (92.0)	369 (95.1)	
IA-FL (*n*, %)	22 (2.4)	13 (2.5)	9 (2.2)	0.79	0 (0.0)	19 (2.4)	10 (2.6)	9 (2.3)	1.00
IAS (*n*, %)	23 (2.5)	15 (2.9)	8 (2.0)	0.37	0 (0.0)	15 (1.9)	8 (2.1)	7 (1.8)	1.00
Coronary malperfusion (*n*, %)	26 (2.8)	19 (3.6)	7 (1.7)	0.07	0 (0.0)	13 (1.7)	6 (1.5)	7 (1.8)	1.00
Cerebral malperfusion (*n*, %)				0.004*	0 (0.0)				0.80
No	882 (94.9)	487 (93.1)	395 (97.3)			757 (97.6)	380 (97.9)	377 (97.2)	
Unilateral	45 (4.8)	35 (6.7)	10 (2.5)			17 (2.2)	7 (1.8)	10 (2.6)	
Bilateral	2 (0.2)	1 (0.2)	1 (0.2)			2 (0.3)	1 (0.3)	1 (0.3)	
SMA-malperfusion (*n*, %)				0.85*	0 (0.0)				0.97
No	777 (83.6)	434 (83.0)	343 (84.5)			659 (84.9)	331 (85.3)	328 (84.5)	
Dynamic	131 (14.1)	78 (14.9)	53 (13.1)			98 (12.6)	48 (12.4)	50 (12.9)	
Static	19 (2.0)	10 (1.9)	9 (2.2)			18 (2.3)	9 (2.3)	9 (2.3)	
Mix	2 (0.2)	1 (0.2)	1 (0.2)			1 (0.1)	0 (0.0)	1 (0.3)	
Renal malperfusion	62 (6.7)	32 (6.1)	30 (7.4)	0.44		59 (7.6)	32 (8.2)	27 (7.0)	0.50
Left lower limb malperfusion (*n*, %)				0.15*	0 (0.0)				0.99
No	876 (94.3)	498 (95.2)	378 (93.1)			730 (94.1)	366 (94.3)	364 (93.8)	
Dynamic	51 (5.5)	25 (4.8)	26 (6.4)			46 (5.9)	22 (5.7)	24 (6.2)	
Static	2 (0.2)	0 (0.0)	2 (0.5)			0	0 (0.0)	0 (0.0)	
Right lower limb malperfusion (*n*, %)				0.61	0 (0.0)				0.78
No	863 (92.9)	489 (93.5)	374 (92.1)			722 (93.0)	362 (93.3)	360 (92.8)	
Dynamic	41 (4.4)	20 (3.8)	21 (5.2)			38 (4.9)	19 (4.9)	19 (4.9)	
Static	25 (2.7)	14 (2.7)	11 (2.7)			16 (2.1)	7 (1.8)	9 (2.3)	

DAC, dual arterial cannulation; SAC, axillary artery cannulation; BMI, body mass index; HT, hypertension; CAD, coronary artery disease; AF, atrial fibrillation; DM, diabetes mellitus; CRI, chronic renal insufficiency; Scr, serum creatinine; TEVAR, thoracic endovascular aortic repair; AVR, aortic valve replacement; AR, aortic regurgitation; aAO, ascending aorta; DTA, descending thoracic aorta; AA, abdominal aorta; IA-FL, innominate artery originating from false lumen; IAS, innominate artery stenosis; and SMA, superior mesenteric artery. *Fisher's exact test was used.

**Table 2:** Operative characteristics.

	**Unmatched**			***P***	**Matched**			***P***
**Variable**	**Overall** ***n =* 929**	**DAC** ***n =* 523**	**SAC** ***n =* 406**		**Overall** ***n =* 776**	**DAC** ***n =* 388**	**SAC** ***n =* 388**	
Aortic root surgery (*n*, %)				0.019				0.07
Supracoronary aortic replacement	667 (71.8)	377 (72.1)	290 (71.4)		564 (72.7)	290 (74.7)	274 (70.6)	
Bentall	241 (25.9)	136 (26.0)	105 (25.9)		193 (24.9)	90 (23.2)	103 (26.5)	
David	9 (1.0)	1 (0.2)	8 (2.0)		8 (1.0)	0 (0.0)	8 (2.1)	
AVR	12 (2.3)	9 (1.7)	3 (0.7)			8 (2.1)	3 (0.8)	
CABG (*n*, %)	113 (12.2)	66 (12.6)	47 (11.6)	0.63	83 (10.7)	38 (9.8)	45 (11.6)	0.41
Femoral artery bypass (*n*, %)	32 (3.4)	22 (4.2)	9 (2.2)	0.35	30 (3.9)	21 (5.4)	9 (2.3)	0.025
Carotid artery bypass (*n*, %)	15 (1.6)	10 (1.9)	5 (1.2)	0.42	13 (1.7)	8 (2.1)	5 (1.3)	0.40
CPB duration, min ($\bar{X}$ ± SD)	195.1 ± 68.1	204.8 ± 62.7	182.7 ± 72.7	<0.001	192.6 ± 70.0	202.2 ± 64.6	183.0 ± 73.9	<0.001
X-clamp duration, min ($\bar{X}$ ± SD)	108.3 ± 33.8	112.9 ± 31.9	102.4 ± 35.3	<0.001	106.6 ± 33.2	110.7 ± 30.3	102.4 ± 35.5	<0.001
HCA duration, min ($\bar{X}$ ± SD)	18.7 ± 6.8	18.8 ± 8.0	18.6 ± 4.8	0.51	18.8 ± 6.6	19.13 ± 8.0	18.5 ± 4.7	0.29
SCP duration, min ($\bar{X}$ ± SD)	27.1 ± 8.5	29.5 ± 8.7	23.9 ± 7.0	<0.001	26.8 ± 8.5	29.7 ± 8.9	23.9 ± 6.9	<0.001
Nasopharyngeal temperature, °C ($\bar{X}$ ± SD)	22.0 ± 3.6	21.6 ± 3.8	22.4 ± 3.2	0.001	21.9 ± 3.5	21.5 ± 3.7	22.4 ± 3.2	<0.001
Rectal temperature, °C ($\bar{X}$ ± SD)	24.9 ± 4.0	24.0 ± 3.9	26.2 ± 3.8	<0.001	25.0 ± 4.0	23.9 ± 3.8	26.1 ± 3.9	<0.001

*DAC, dual arterial cannulation; SAC, axillary artery cannulation; AVR, aortic valve replacement; CABG, coronary artery bypass grafting; CPB, cardiopulmonary bypass; HCA, hypothermic circulatory arrest; and SCP, selective cerebral perfusion*.

### Perioperative Outcomes

In the whole cohort, 57 patients (6.1%) died, and the main presumed causes of death were heart-related circulatory failure, acidosis induced by visceral malperfusion, and stroke. Stroke and spinal cord injury occurred in 57 (6.1%) and 49 (5.3%) patients, respectively. In total, 111 (11.9%) patients had acute renal failure and required CRRT. Operative mortality was similar between the 2 groups (6.7 vs. 5.4%, *P* = 0.42 before matching; 5.4 vs. 5.4%, *P* = 1 after matching). Unadjusted and adjusted risks of stroke were similar across the initial cannulation sites (6.7 vs. 5.4%, *P* = 0.42 before matching; 6.4 vs. 5.2%, *P* = 0.44 after matching). Spinal cord injury was also distributed similarly in the two groups (5 vs. 5.7%, *P* = 0.64 before matching; 5.4 vs. 5.7%, *P* = 1 after matching). A trend that CRRT was more frequent in the DAC group was observed (13.8 vs. 9.6%, *P* = 0.05), but the trend disappeared after matching (12.6 vs. 9.5%, *P* = 0.17). The incidences of other complications were similar between the 2 groups whether before or after matching. The perioperative outcomes were listed in Table 3.

**Table 3:** Perioperative outcome characteristics.

	**Unmatched**			***P***	**Matched**			***P***
**Variable**	**Overall** ***n =* 929**	**DAC** ***n =* 523**	**SAC** ***n =* 406**		**Overall** ***n =* 776**	**DAC** ***n =* 388**	**SAC** ***n =* 388**	
MV duration, hour (median, IQR)	22.0 (14.0–56.0)	24.0 (14.0–64.0)	20.0 (14.0–45.0)	0.019*	22.0 (14.0–50.5)	24.0 (14.0–61.0)	20.0 (14.0–44.5)	0.038*
ICU stay, day (median, IQR)	3.0 (2.0–6.0)	4.0 (2.0–6.0)	3.0 (2.0–5.0)	<0.001*	3.0 (2.0–6.0)	4.0 (2.0–6.0)	3.0 (2.0–5.0)	0.002*
Operative mortality (*n*, %)	57 (6.1)	35 (6.7)	22 (5.4)	0.42	42 (5.4)	21 (5.4)	21 (5.4)	1.00
PMI (*n*, %)	4 (0.4)	3 (0.6)	1 (0.2)	0.64^†^	3 (0.4)	2 (0.5)	1 (0.3)	1.00
Stroke (*n*, %)	57 (6.1)	35 (6.7)	22 (5.4)	0.42	45 (5.8)	25 (6.4)	20 (5.2)	0.44
Spinal cord injury (*n*, %)	49 (5.3)	26 (5.0)	23 (5.7)	0.64	43 (5.5)	21 (5.4)	22 (5.7)	1.00
Reoperation for bleeding (*n*, %)	41 (4.4)	21 (4.0)	20 (4.9)	0.50	38 (4.9)	18 (4.6)	20 (5.2)	0.87
IABP (*n*, %)	3 (0.3)	1 (0.2)	2 (0.5)	0.58^†^	3 (0.4)	1 (0.3)	2 (0.5)	1.00
ECMO (*n*, %)	6 (0.6)	1 (0.2)	5 (1.2)	0.09^†^	6 (0.8)	1 (0.3)	5 (1.3)	0.22
CRRT (*n*, %)	111 (11.9)	72 (13.8)	39 (9.6)	0.05	86 (11.1)	49 (12.6)	37 (9.5)	0.17
Tracheotomy (*n*, %)	24 (2.6)	15 (2.9)	9 (2.2)	0.53	21 (2.7)	12 (3.1)	9 (2.3)	0.66
Intestinal ischemia (*n*, %)	12 (1.3)	8 (1.5)	4 (1.0)	0.46	9 (1.6)	5 (1.3)	4 (1.0)	1.00

DAC, dual arterial cannulation; SAC, axillary artery cannulation; MV, mechanical ventilation; IQR, Interquartile Range; ICU, intensive care unit; PMI, perioperative myocardial infarction; IABP, intra-aortic balloon pump implantation; ECMO, extracorporeal membrane oxygenation; and CRRT, continuous renal replacement therapy. *Wilcoxon signed rank test was used.^†^Fisher's exact test was used.

### Multivariate Logistic Regression Analysis and Stratification Analysis

**Figure 1:**
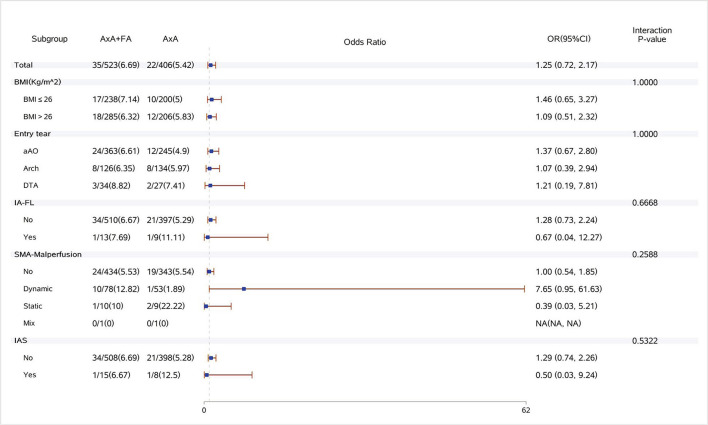
Forest plot of dual arterial cannulation vs. axillary artery cannulation regarding operative mortality by subgroups. AXA+FA, axillary artery cannulation+femoral artery cannulation; AXA, axillary artery cannulation; IA-FL, innominate artery originating from false lumen; SMA, superior mesenteric artery; and IAS, innominate artery stenosis.

**Figure 2:**
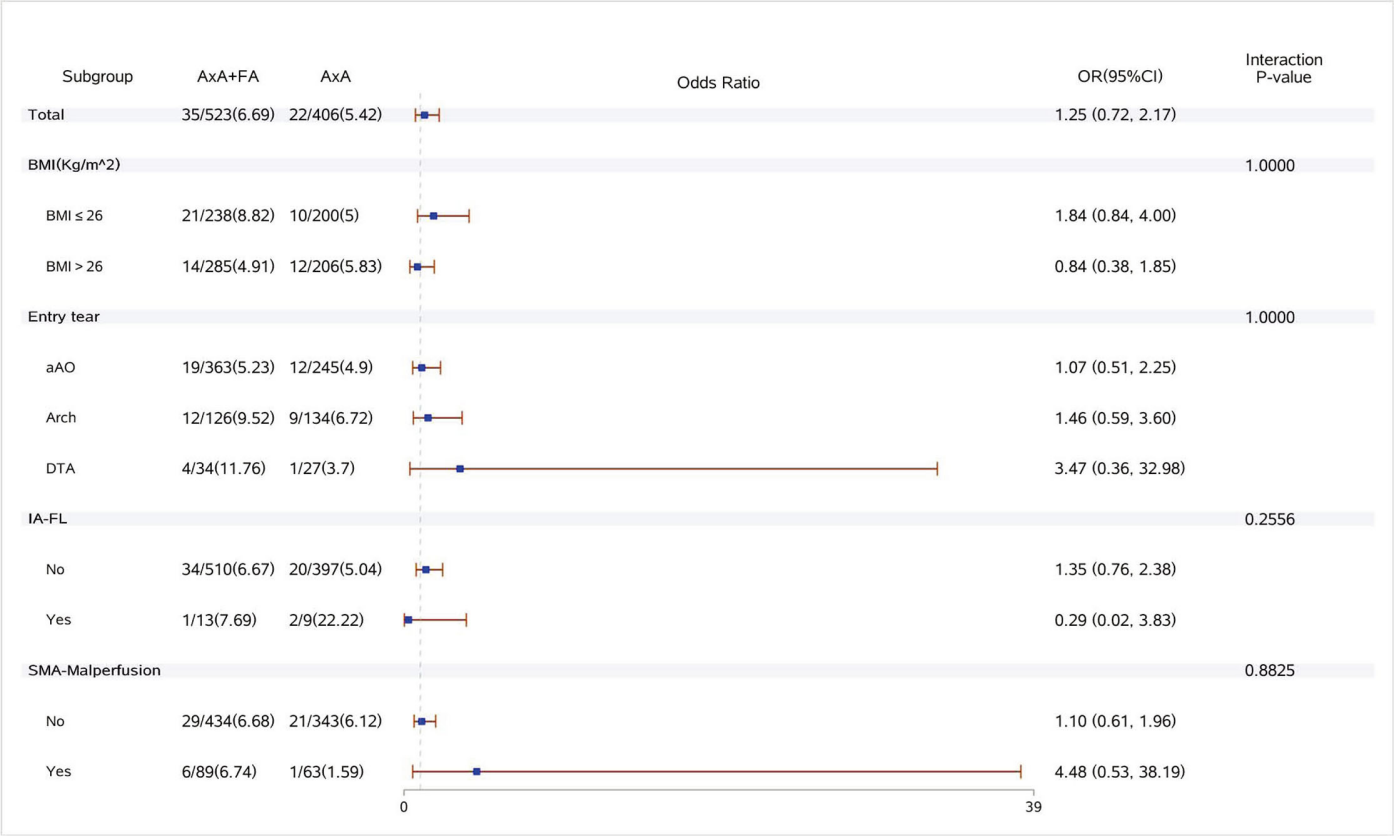
Forest plot of dual arterial cannulation vs. axillary artery cannulation regarding stroke by subgroups. AXA+FA, axillary artery cannulation+femoral artery cannulation; AXA, axillary artery cannulation; IA-FL, innominate artery originating from false lumen; and SMA, superior mesenteric artery.

**Figure 3:**
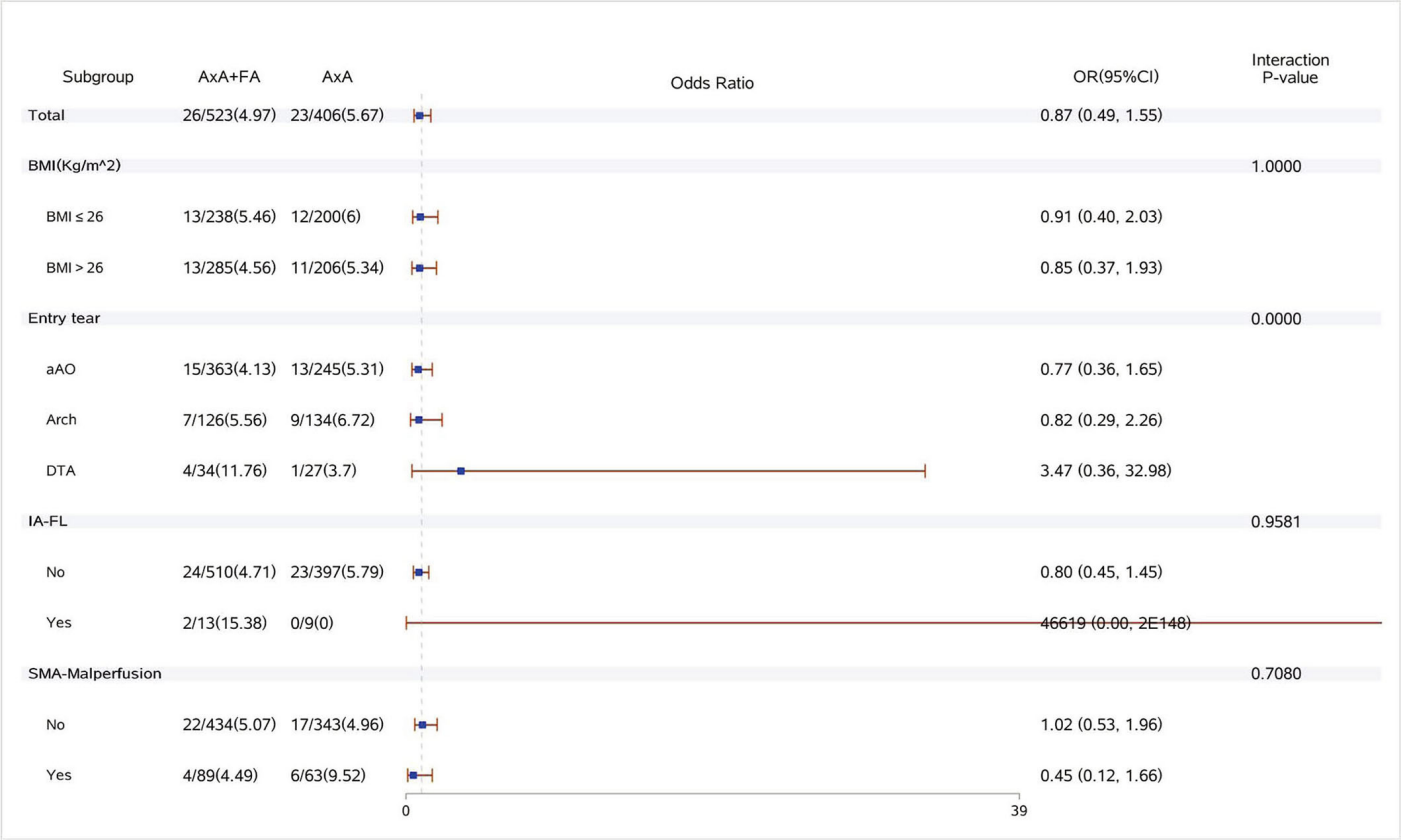
Forest plot of dual arterial cannulation vs. axillary artery cannulation regarding spinal cord injury by subgroups. AXA+FA, axillary artery cannulation+femoral artery cannulation; AXA, axillary artery cannulation; IA-FL, innominate artery originating from false lumen; and SMA, superior mesenteric artery.

**Figure 4:**
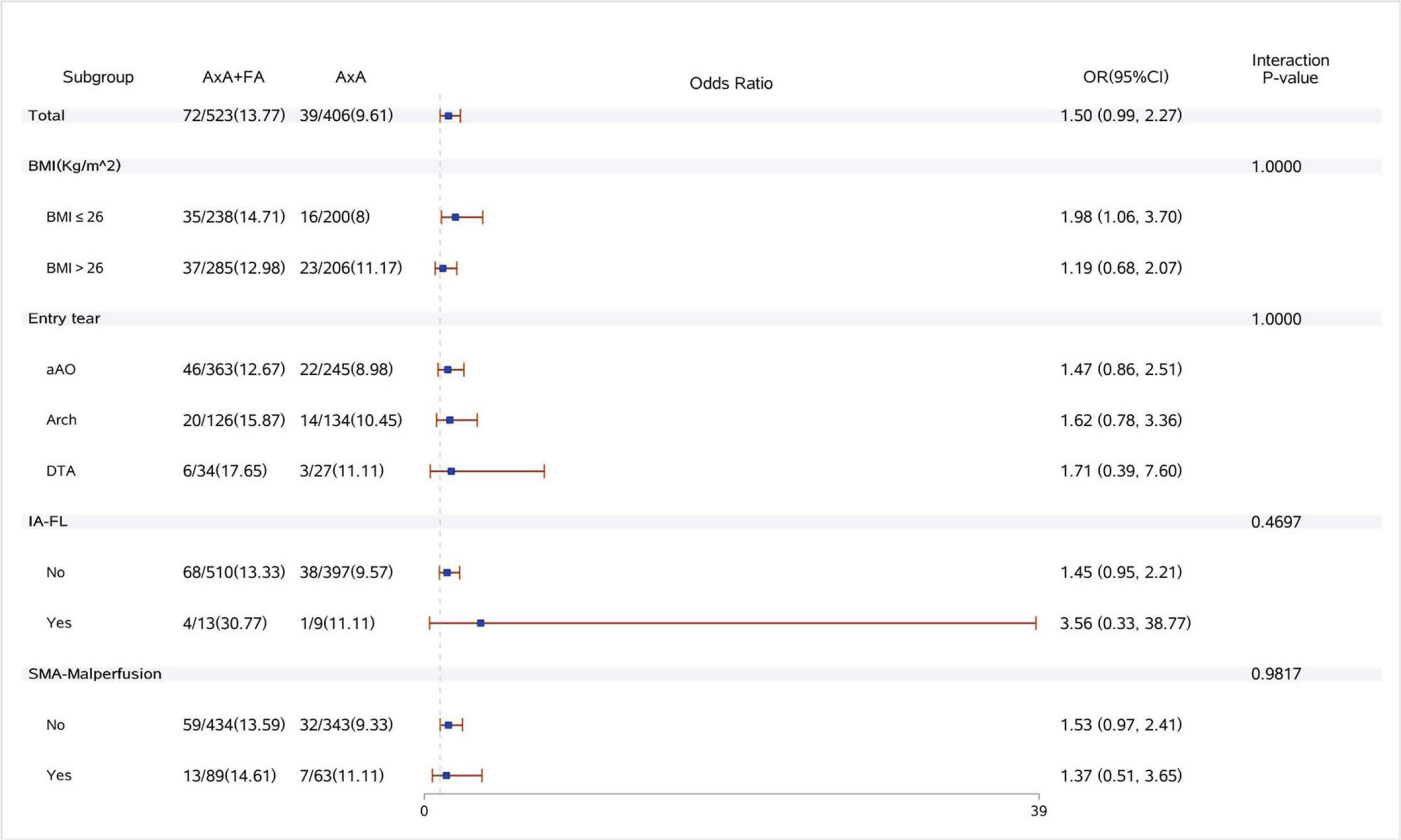
Forest plot of dual arterial cannulation vs. axillary artery cannulation regarding acute renal failure requiring dialysis by subgroups. AXA+FA, axillary artery cannulation+femoral artery cannulation; AXA, axillary artery cannulation; IA-FL, innominate artery originating from false lumen; and SMA, superior mesenteric artery.

Dual arterial cannulation was not an independent protective factor of operative mortality (odds ratio [OR] 1.01, 95% confidence interval [CI] 0.55–1.86), stroke (OR 1.17, 95% CI 0.65–2.11), spinal cord injury (OR 1.17, 95% CI 0.65–2.11), and acute renal failure requiring CRRT (OR 1.24, 95% CI 0.78–1.97) after adjusting for confounding factors by multivariable logistic regression analysis (see detailed results of three models in Table 4). After stratification according to BMI, dual arterial cannulation was associated with a significantly higher risk of CRRT in patients with a body mass index (BMI) of ≤ 26 (OR, 1.98; 95% CI 1.06–3.7). In other subgroups of entry tear location, innominate artery originating from false lumen, superior mesenteric artery malperfusion, and innominate artery stenosis, and dual arterial cannulation had no significant advantage over a single axillary artery cannulation on the primary outcomes. The incidence of stroke, spinal cord injury, and CRRT in the subgroup of IAS was low or even 0, making stratification results meaningless so that subgroup analysis of IAS was conducted only for operative mortality. The results of subgroup analysis were demonstrated in Figures 1–4.

**Table 4:** Multivariable analyses of operative mortality, stroke, spinal cord injury, and acute renal failure requiring CRRT for dual arterial cannulation vs. single axillary artery cannulation.

	**Odds ratio (95% CI)**	***P* value**	**Reference**
Operative mortality			Axillary cannulation
Model 1	1.06 (0.58, 1.92)	0.85	
Model 2	1.04 (0.57, 1.9)	0.89	
Model 3	1.01 (0.55, 1.86)	0.98	
Stroke			Axillary cannulation
Model 1	1.11 (0.62, 1.96)	0.73	
Model 2	1.12 (0.63, 1.99)	0.70	
Model 3	1.17 (0.65, 2.11)	0.60	
Spinal cord injury			Axillary cannulation
Model 1	0.83 (0.45, 1.5)	0.53	
Model 2	0.85 (0.46, 1.54)	0.59	
Model 3	1.17 (0.65, 2.11)	0.60	
ARF-CRRT			Axillary cannulation
Model 1	1.17 (0.75, 1.81)	0.49	
Model 2	1.18 (0.76, 1.84)	0.46	
Model 3	1.24 (0.78, 1.97)	0.36	

*OR, odds ratio; CI, confident interval. Model 1: adjusted for age/CAD/previous stroke/coronary malperfusion/cerebral malperfusion/superior mesenteric artery malperfusion/hemodynamic instability/CPB duration/preoperative serum creatinine. Model 2: adjusted for age/BMI /CAD/EF/previous stroke/chronic renal insufficiency/ aortic regurgitation>moderate/coronary malperfusion/ cerebral malperfusion/superior mesenteric artery malperfusion/hemodynamic instability/CPB duration/preoperative serum creatinine. Model 3: adjusted for age/BMI /CAD/EF/previous stroke/chronic renal insufficiency/ aortic regurgitation>moderate/coronary malperfusion/ cerebral malperfusion/superior mesenteric artery malperfusion/hemodynamic instability/CPB duration/preoperative serum creatinine/left lower limb malperfusion/right lower limb malperfusion*.

## Discussion

The present study is important because it compares two cannulation strategies in the context of varying complexity of acute type A aortic dissection. The main findings of our study can be summarized as follows:

1. Regarding pathological features of dissection, single axillary cannulation was comparable with dual arterial cannulation for complex arch repair in the acute type A aortic dissection.
2. Based on antegrade perfusion, adding femoral artery cannulation did not result in an elevated rate of stroke caused by a retrograde blood flow.

Total arch replacement (TAR) combined with FET could provide a stable distal anastomosis and ideal remodeling of the downstream aorta, therefore, it was appropriate for patients with extensive dissection, presenting with satisfactory early and long-term results (5, 6, 8). Overall, compared with recent studies (6, 7), we had a lower operative mortality and a comparable incidence of main complications in the present study. The SCP time of the single axillary group was 5 min faster than that of the dual arterial group, in fact, the dual cannulation group mainly consisted of the patients who received operation in the earlier stage, and the single axillary cannulation group mainly consisted of the patients in the later stage. We became more skillful and the SCP time was shorter in the later stage. A higher incidence of spinal cord injury was found in our study. The FET products we used had only two length specifications: 100 mm and 120 mm. In most cases, we preferred a 120 mm FET in order to achieve a better remodeling of the distal aorta. The FET depth, which varied with body height, ranged from T5-T8 vertebral body. It was well-discussed that an intercostal artery originating from false lumen is a risk factor of spinal cord injury besides application of FET. So, we speculated that longer FETs for patients who were at risk of SCI were responsible for the high incidence of SCI in our cohort.

Restoring organ perfusion was critical in the early stage of CPB because pulsatile blood flow attenuated and even disappeared. The arterial cannulation site was an important issue of the whole procedure. Many types of research (9–12) were conducted to discover the optimal cannulation strategy, drawing an identical or conflicting conclusion. Axillary artery cannulation providing antegrade perfusion was advocated by more surgeons. The advantages and limitations of axillary artery cannulation vs. femoral artery cannulation were well-discussed in previous studies (13, 14). Some investigators attempted to apply a dual arterial cannulation to overcome their respective drawbacks (15, 16). It was considered reasonable that morphologic variability of dissection might interplay with cannulation and affect outcomes (17). That is why the cannulation site would possibly make a difference in clinical outcomes, although it worked for a short period of time (usually less than 1 h) from the establishment of CPB to the initiation of hypothermic circulatory arrest. But, this point was seldom taken into full consideration when the cannulation strategy was investigated in previous studies. In our study, we adjusted the preoperative comorbidities and special pathological features of aortic dissection by propensity-score matching. Judging from the statistical results, single axillary artery cannulation was competent in a complex aortic dissection regarding primary outcomes. According to the analysis of some investigators (18–20), persistent false lumen perfusion and true lumen collapse after initiation of CPB might lead to an exacerbation of pre-existing malperfusion or a newly emerging malperfusion. Theoretically, a persistent false lumen perfusion occurred more frequently in patients using a retrograde blood flow through femoral artery cannulation (18, 19); and if entry tear is located farther distally (in or beyond the transverse arch) or an innominate artery is originated from false lumen, the same phenomenon might occur when axillary artery cannulation was applied. As Orihashi et al. (18) reported, false lumen perfusion was detected in 8.5% of cases while axillary artery cannulation was used. Lin and coworkers (15) reported that dual arterial cannulation had significant advantages on hospital mortality, stroke, and malperfusion-related complications over a single arterial cannulation. In their single arterial cannulation group, femoral artery cannulation was predominant so that the gap in results might be exaggerated. In our cohort, either operative mortality or incidence of main complications was similar between the two groups. When the innominate artery was involved, axillary artery cannulation was also safe, this finding was similar to previous studies (21, 22). Regarding malperfusion-related complications, such as acute renal failure and intestinal ischemia, there was still no significant difference. The statistical results of the three models were consistent no matter how many variables were adjusted. In stratification analysis, for patients with innominate artery originating from the false lumen and distal entry tear, the single axillary artery cannulation group did not have disadvantages on primary outcomes. On the other hand, there were still some studies showing comparable results of single femoral artery cannulation without a raised risk of malperfusion-related complications (13, 14). According to these findings, it can be hypothesized that a persistent false lumen perfusion might be a random effect and is not as common as Orihashi et.al reported (18). As Rosinski proposed (9): “outcomes are largely determined according to patient presentation rather than cannulation site”. To prevent over-interpretation of the results, a detailed monitoring and fluid dynamics analysis were needed to explain the mechanism.

Indeed, whether using additional cannulation should base on intraoperative findings. Firstly, if high-flow resistance occurred when a single axillary artery was used, an extra femoral artery cannulation was necessary (9, 23). In fact, we seldom encountered insufficient flow volume *via* single axillary artery cannulation because patients in our cohort had a relatively small body mass index. Even single axillary artery cannulation was competent in 8 patients with innominate artery stenosis. Secondly, if malperfusion was detected after initiation of CPB, shifting or adding cannulation should be considered (17–19). Peripheral arterial blood pressure and near-infrared spectroscopy were available in most institutes. Transesophageal echocardiography and orbital Doppler could be used to detect intraoperative superior mesenteric artery and cerebral malperfusion (18). Even though it is not easy to implement multiple monitoring methods simultaneously, we still believe that an accurate monitoring can avoid strategic blindness.

This study has some important limitations. Firstly, it is a retrospective study and selection bias might exist although a propensity-score matching partially made up for the deficiency. Secondly, we did not use a direct intraoperative surveillance to evaluate malperfusion during CPB. Third, cases with adverse events were very few in subgroups and statistical analysis was of limited significance.

## Conclusion

Single axillary artery cannulation was competent in most cases, presenting with a satisfactory result as dual arterial cannulation. Adding femoral artery cannulation was necessary when the sufficient flow volume could not be achieved by axillary artery cannulation or lower limb malperfusion existed. Under antegrade blood flow, femoral artery cannulation would not increase the stroke risk.

## Data Availability Statement

The datasets presented in this article are not readily available because institutional policy restriction. Requests to access the datasets should be directed to Yi Chang, chantlinguish@163.com.

## Ethics Statement

The studies involving human participants were reviewed and approved by The Ethics Committee of Fuwai Hospital. Written informed consent for participation was not required for this study in accordance with the national legislation and the institutional requirements.

## Author Contributions

YC was responsible for the conceptualization, data curation, statistical analysis, and writing—original draft. HL was responsible for the statistical analysis. XQ was responsible for the conceptualization, methodology, investigation, and funding acquisition. HG, CY, XS, BW, QM, YW, and YS were responsible for the investigation. All authors contributed to the article and approved the submitted version.

## Funding

This work was supported by CAMS Innovation Fund for Medical Sciences (CIFMS) 2020-I2M-C&T-A-011.

## Conflict of Interest

The authors declare that the research was conducted in the absence of any commercial or financial relationships that could be construed as a potential conflict of interest.

## Publisher's Note

All claims expressed in this article are solely those of the authors and do not necessarily represent those of their affiliated organizations, or those of the publisher, the editors and the reviewers. Any product that may be evaluated in this article, or claim that may be made by its manufacturer, is not guaranteed or endorsed by the publisher.
