# Supplementary material for: Comparison of Single Axillary vs. Dual Arterial Cannulation for Acute Type a Aortic Dissection: A Propensity Score Matching Analysis
Source: Front Cardiovasc Med. 2022 Feb 23;9:809493. doi: 10.3389/fcvm.2022.809493 (PMC8904879; doi:10.3389/fcvm.2022.809493)
Supplement: Supplementary file 1 [file Data_Sheet_1.PDF]

SUPPLEMENTAL MATERIAL

Expanded Methods -Indications and surgical technique of total arch replacement combined with frozen elephant trunk

Indications

(1) primary tear locating in the transverse arch or the descending aorta; (2) aortic arch was involved totally; (3) aneurysm formation in aortic arch or distal aorta (aneurysm size 45 mm); (3) involvement of, aneurysm formation in, and occlusion of the brachiocephalic artery; or (4) connective tissue disease, such as Marfan syndrome. If aortic dissection extended beyond the distal arch, a frozen elephant trunk (FET) was implanted into the distal aorta.

Surgical technique

After cardiopulmonary bypass (CPB) established, ascending aorta was clamped and cardioplegia was transmitted to coronary orifice directly. Aortic root repair was done during cooling phase. Circulatory arrest was instituted if the nasopharyngeal temperature reached target temperature. The supra-arch arteries were clamped and the aortic arch was open. Selective cerebral perfusion (SCP) was started through the right axillary artery. Aortic arch was resected between the origin of the left subclavian artery and the left carotid artery and supra-arch arteries were resected at their initial part. The FET was deployed into the true lumen of distal aorta. The distal aorta incorporating the stent graft was sewn to the distal end of a 4-branch prosthetic graft using distal first technique. After completion of distal anastomosis, the graft was cross-clamped, and antegrade systemic perfusion was achieved through a side branch.

The sequence of supra-arch arteries anastomosing to prosthetic graft branches was carried out from left common carotid artery, left subclavian artery and innominate artery. Sometimes innominate artery was anastomosed after proximal aortic stump anastomosis was completed. Usually after the anastomosis of left common carotid artery was accomplished, SCP was discontinued, CPB gradually resumed to normal flow, and rewarming started.

Supplemental Figure I

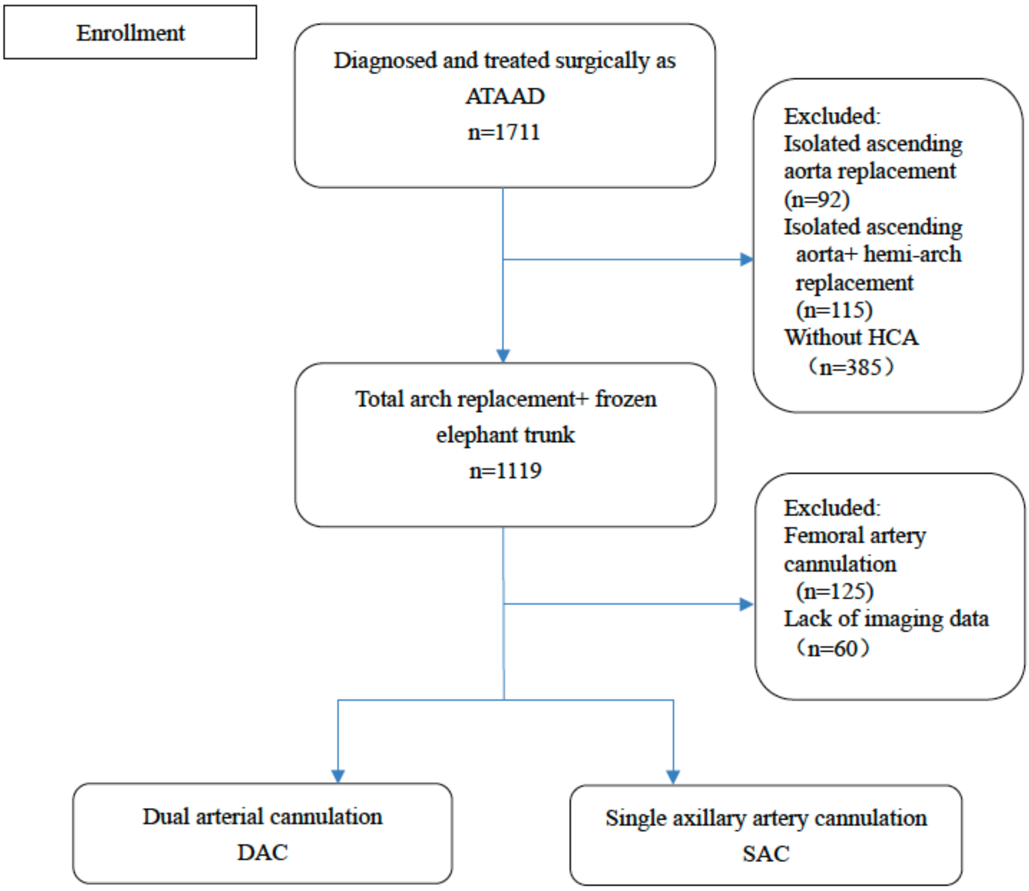

Supplementary Figure I Flow chart of patient enrollment
ATAAD, acute type A aortic dissection; HCA, hypothermic circulatory arrest.

Supplementary Table I Propensity Score Model AUC Analysis and Hosmer Lemeshow Test Result

Index	Model AUC	Model AUC (95%CI)	Model AUC (P Value)	Hosmer Lemeshow Chi-Square Value	Hosmer Lemeshow Test P Value
Propensity Score Model	0.6239	(0.5882, 0.6596)	<0.0001	11.0736	0.1976

Propensity Score model was constructed in 929 patients. Adjusting: age, gender, BMI, hypertension, coronary artery disease, atrial fibrillation, Marfan syndrome, previous stroke, chronic renal insufficiency, previous heart surgery, EF, aortic regurgitation>moderate, hemodynamic instability, entry tear location, coronary malperfusion, cerebral malperfusion, innominate artery originating from false lumen, innominate artery stenosis, superior mesenteric artery malperfusion, left lower limb malperfusion, light lower limb malperfusion. The Propensity Score model was constructed to obtain the probability of each subject being assigned to dual arterial cannulation group, and then the weight of each subject was calculated (dual arterial cannulation weight=1/probability, axillary artery cannulation weight=1/(1-probability)). The analysis results of basic parameters of Propensity Score model are listed in the table.

1.AUC P Value <0.05 is considered that the prediction is significant

2.Calibration (HL test) quantifies how close the predicted probabilities match the actual experience

3.HL test low p values represent lack of fit

Supplementary Table II Absolute standardized differences between the two groups regarding baseline characteristics

Variable	SD before matching	SD after matching
Age, year	0.113	0.007
BMI, Kg/m^2	0.091	0.009
HT	0.042	0.005
CAD	0.079	0.011
AF	0.070	0.000
Marfan syndrome	0.005	0.010
Previous AVR	0.090	0.027
Previous other heart surgery	0.108	0.042
DM	0.068	0.048
Previous stroke	0.053	0.062
CRI	0.047	0.000
EF	0.088	0.014
AR>moderate	0.017	0.042
Entry tear-arch	0.198	0.045
Entry tear-DTA	0.006	0.049
Extent-To arch	0.131	0.131
Extent-To DTA	0.011	0.000
Coronary malperfusion	0.118	0.020
Cerebral malperfusion -Unilateral	0.203	0.053
Cerebral malperfusion -Bilateral	0.012	0.000
IA-FL	0.018	0.017
IAS	0.059	0.019
SMA-malperfusion-Dynamic	0.054	0.016
SMA-malperfusion-Static	0.021	0.000
SMA-malperfusion-Mix	0.012	0.072
Left lower limb malperfusion-Dynamic	0.071	0.022
Left lower limb malperfusion-Static	0.100	NA[#]
Right lower limb malperfusion-Dynamic	0.065	0.000
Right lower limb malperfusion-Static	0.002	0.036

SD, standardized difference; BMI, Body Mass Index; HT, hypertension; CAD, coronary artery disease; AF, atrial fibrillation; DM, diabetes mellitus; CRI, chronic renal insufficiency; AVR, aortic valve replacement; AR, aortic regurgitation; DTA, descending thoracic aorta; IA-FL, innominate artery originating from false lumen; IAS, innominate artery stenosis; SMA, superior mesenteric artery
